# Mr. Bedingfield's Compendium of Medical Practice, &c.

**Published:** 1816-09

**Authors:** 


### *Mr.* Bedingfield's *Compendium of Medical Practice, &c.*

( Continued from page 115 )

### Sect. III. Chap. 2. *Hydrops Pericardii,* p. 134-139.

Mr. B. considers hyd. pericard. as almost always resulting from organic disease of the heart or great vessels. He confesses, that "to distinguish hydrops pericard. from hydrothorax is *extremely* difficult, in some cases impossible. It *frequently* happens that they are united. An operation is not admissible." How does this tally with his urgent proposal for the operation in hydrothorax?

*Case.* A woman, 29 years of age, admitted the 27th of June, 1811.

*Symptoms.* Incapable of supporting herself; depression of spirits; indifference to questions; had been ill some months; took several medicines. Fœtid breath; gums destroyed by mercury; said she had the dropsy; a circumscribed tumor of the abdomen induced a suspicion of pregnancy. Could not deny nor confirm this suspicion. Catamenia stopped for seven months. The warm bath. Pulv. jalap. ℈j; gargle for her mouth.

28th. Skin rather dry and hot; tongue foul; pulse 90; somewhat oppressed, but regular; breathing rather difficult.

" Upon examining the abdomen, a circumscribed tumor was found extending upwards from the pubes to mid-way between the umbilicus and scrobiculus cordis. This tumor was extremely firm, and communicated the sensation of a solid body to the touch. It was endeavoured, but in vain, to distinguish a fluid. The urine was evacuated freely, regularly, and in large quantity. She complained of her mouth very much, but felt no pain in any part of her body."

All these circumstances led to the conclusion that the woman was in the seventh or eighth month of utero-gestation. She was discharged, and died in the course of the following week.

Mr. B. only learnt a few of the post mortem appearances, as the gentleman means to publish the case. Ulcerations, however, of considerable extent, were found on the surface of the heart, and nine ounces of serum were contained in the pericardium. The tumor of the abdomen was occasioned by an enormously enlarged and distended bladder. Upwards of two gallons of urine were discharged upon an incision being made into its parietes. The case is certainly very remarkable, though not so much so as the case of hydro-renal distension lately related in this Journal.

CHAP. 3. *Ventriculorum Cordis Inflammatio.*—Palpitations of the heart have lately become very prevalent. Nearly as many cases of increased action of the heart as of phthisis have been admitted into the Bristol Infirmary within the last five years.

" Scarcely a female applies for the removal of hysterical symptoms, or labouring under amenorrhœa, but, in addition, she is affected with increased and inordinate action of the heart and carotid arteries. Amongst men, likewise, this affection is become very common. The metastasis of rheumatic inflammation from the extremities to the heart, often gives rise to it, particularly when venæsection has been omitted, or but sparingly employed."

*Case.* William Trick, ætat. 25, applied for relief January 10th, 1811. States his having been ill six months; conceives his complaint was brought on by violent exertion in a coal-pit, when he was seized with cough, spitting of blood, &c.; since which, palpitation of the heart, difficulty of breathing, cough, decrease of strength, and emaciation, have continued in despite of medicine. The physician of the week pronounced it organic affection of the heart; incurable. A saline antimonial medicine and lohoc for the cough.

14th. He returned with an aggravation of all the symptoms. Scarcely able to walk; breathing very laborious; violent palpitatio cordis; pulse hurried, irregular, intermittent; extreme anxiety of countenance. A grain of opium, and to be repeated *pro re nata. Six in the evening.* Heart throbbing against the parietes of the chest with astonishing velocity, irregularity, and unprecedented violence. Pulsations of the radial artery innumerable; breathing extremely laborious. Twelve ounces of blood from the external jugular seemed to afford some relief; breathing less difficult; pulse more regular. Capiat tinct. opii ʒj. Died at two in the morning.

*Sectio Cadaveris.* Pericardium healthy, no effusion in its cavity; heart rather large, coated at its basis, and upon its under surface with more than usual adipose substance. Auricles healthy; inner surface of the ventricles highly vascular; tricuspid and mitral valves evidently inflamed; outer coats of the aorta and pulmonary artery rather red; internal linings of ditto looked precisely like a highly inflamed tunica conjunctiva. No other morbid appearances. Might not copious bleedings have afforded some prospect of success here?

### CHAP. 4. *On the Velocity and Inequality of the Action of the Heart.*

*Case.* John Ferrol, ætat. 23, by trade a cooper, has been indisposed about twelve months. His symptoms at present (16th December, 1811) are an attack of *palpitatio cordis* every morning and evening, the heart acting with great force and rapidity, pulsating 212 times in the minute. During this increased action, no pulse at the wrist, nor was the action of the carotids very evident. No pain, but constant dread of suffocation; breathing very laborious; countenance bloated, and of a dark blue colour. In about an hour these phænomena would be changed for others still more curious. The radial artery would strike the finger about 70 times in the minute, while the heart would beat distinctly against the parietes of the chest exactly double that number. This state of the pulse, &c. would continue also about an hour, when the heart and arteries would move in unison about 95 times in the minute. The evening paroxysms were more distressing than those of the morning: the duration of both could be somewhat shortened by exhibiting a drachm of æther with twenty drops of laudanum. He took, for a fortnight, a saline mixture with a small quantity of digitalis; a pill, composed of one grain of calomel, and three of powdered squill, every morning, and ten grains of pulv. ipec. comp. every night. A cathartic occasionally. By this treatment he was somewhat relieved; but prepossessed with an idea, that in the event of dying he should be opened, he left the house, and died about three weeks afterwards.

In some comments on this singular case, Mr. B. anathematises a medical gentleman at Bristol, who doubted the truth of his statement respecting the enumeration of the pulses up to 212. We acknowledge that it is possible to *count* 212 in the minute; but we are much inclined to think, that 212 pulsations could not be *felt* and *enumerated* from this feeling in the short space of one minute. Medical authors, however, are divided in opinion on this subject, and Mr. B. might have quoted the authority of WENDT (De Pulsus mutatione insigni) for the possibility of enumerating 243 strokes in the above-mentioned space of time. On the nature of the cardiac lesion, which gave rise to those curious phænomena, we shall offer no opinion, since it could be grounded on conjecture only.

The fifth Chapter of this Section, on *Polypi of the Heart*, offers nothing new, except the hint of *scorbutising* the system as a *preventive!* We will not go so far as to say that this would be a *silly* trick, but, certes, it would be a *scurvy* one.

CHAP. 6. *Aneurism of the Aorta.*

*Case.* Mary Townsend, ætat. 30, emaciated, affected with cough, pain in the left side, violent palpitation of the heart, was admitted on the 24th of February, 1814. She was sometime a week or more free from these symptoms, but in general had one or two attacks daily, which could be moderated by æther and opium, as in the case before related. When the respiration was laborious, a few ounces of blood were occasionally taken from the arm. The pulse during the paroxysm was rapid and indistinct; afterwards, soft, regular, and about 80 in the minute.

Many remedies were prescribed, particularly small doses of mercury, opium, digitalis, æther, camphor, venæsection, &c. She appeared once or twice to be nearly recovered, but upon using exertion the palpitation returned.

Three days before dissolution, after walking out, she was seized with great pain in the chest, laborious respiration, and a slight hæmorrhage from the lungs. Venæsection to ℥xij, and an opiate at night. The day preceding death, she coughed up nearly a quart of blood; became faint; cessation of pulse at the wrist; violent throbbing of the cervical vessels. Infus. rosæ acid. opiate at bed-time. At ten o'clock next day, the hæmorrhage returned: the blood gushed in a full and uninterrupted stream from the mouth, till nearly five pints were evacuated, when she sunk back and expired.

*Sectio cadaveris.*—The stomach, instead of stretching obliquely across the upper part of the abdominal cavity, was placed longitudinally, or parallel with the linea alba. Its cardiac extremity was opposed to the diaphragm, its pyloric nearly reached the brim of the pelvis. It contained large pieces of coagulated blood, mixed with a coffee-coloured fluid. An aneurism of the arch of the aorta had burst into the lungs. The liver had no left lobe.

CHAP. 7. *The Functions of the Pulmonary Artery.*—The brilliant discovery which this Chapter contains will not detain us long. It is a *conjecture,* without a single experiment or proof, that "The power of converting chyle into blood is resident in the pulmonary artery." In short, we are here told, that the blood itself is *secreted* in the pulmonary artery.

"Every part of the body, whether solid or fluid, is the product of secretion. Is it probable, that so important a fluid as blood should be without its appropriate organ for secretion? The centrical situation of the pulmonary artery, its proximity to the thoracic duct, and to the aorta, renders it peculiarly eligible for the *purpose of secreting blood.*" 168.

Where, we would ask, is the blood *secreted* during the fœtal state, when the pulmonary artery is next to a nonentity?

—Again, has Mr. B. examined the blood of the pulmonary artery and vein, to shew that chyle exists in the former and not in the latter? Alas, no. But there is no necessity for experiment; we have only to think the theory is true, and it is so! We are at a loss, however, to know the "immense importance" of this discovery, even were it proved, that the chyle is converted into blood by the pulmonary artery. Would a knowledge of this lessen the number of hectics which annually meet our view?

Section IV. Chap. 1. *Dyspepsia.*—We agree with Mr. B. that "dyspeptic symptoms will be found, for the most part, to arise from derangement in the structure or functions of the brain, liver, pancreas, uterus, and kidnies;" and consequently, the source of the malady is to be ascertained if possible.

*Case.* Thomast Bust, ætat. 29, by trade a painter, was admitted for colica pictonum, with paralysis of the muscles of the fore-arms, and an excessive irritability of stomach. After constipation was removed, a variety of remedies were tried, which rather aggravated than relieved the *cardialgia*, nausea, and sickness, with which he was perpetually tormented. The stomach rejected every thing *unchanged.* Five grains of the blue pill, with half a grain of opium, taken every night for a week, enabled the stomach to retain food. In a fortnight the gums were affected, the dyspeptic symptoms were nearly removed, and the wrists were evidently stronger. In three weeks, the dyspeptic symptoms were entirely gone, and the muscles were obedient to the will. Pyrosis is treated by the blue pill also.

Chap. 2. *Hæmatemesis.*—There is nothing worthy of notice in this short chapter of a page and a half.

Chap. 3. *Ulceration of the Stomach.*—The pyloric extremity of the stomach is very liable to scirrhus and ulceration—the most terrible diseases to which the human frame is liable, since we can but rarely even palliate the sufferings of the wretched victim.

*Case.* A poor emaciated creature, 52 years of age, died in the Infirmary, after two years of excruciating pain in the stomach, attended sometimes with a total inability to retain food.

*Sectio cadaveris.*—The stomach adhered to the liver; more than one third of its inner surface was in a state of ulceration.

"At its upper part its coats had been entirely removed, and an extensive cavity made in the substance of the liver. The excavated liver truly formed at this place a substitute for the stomach. The pyloric extremity of this organ was much thickened, and its opening into the duodenum nearly closed."

CHAP. 4. *Gastritis.*—The insidious nature of this disease is illustrated by the following case.

H. H. ætat. 20, was admitted 28th of August, 1815, affected with slight pyrexia, but no local pain, nausea, nor sickness. No epigastric nor abdominal tenderness on pressure. Cathartic; saline medicines.

29th.--30th. Same state.—31st. Limbs swollen and painful; in short, all the symptoms of acute rheumatism. Cathartic. Venæsectio ad ℥xvi. Blood buffy.

September 1st. Much relieved, but pulse continues full and frequent. Venæs. ad ℥xvj; cupped and buffed.

Sept. 2d. A most extraordinary and inexplicable alteration had taken place; facies hippocratica; tongue dry and brown; skin covered with a cold clammy perspiration; pulse scarcely perceptible. In short, he exhibited the picture of typhus gravior in its ultimate stage! Death in a few hours.

*Sectio cadaveris.* Traces of inflammation over the whole surface of the peritonæum, extending in some places to the coats of the intestines. Pelvis full of serum, and flakes of coagulable lymph. Small intestines glued together. Stomach remarkably small, but apparently sound externally. Nearly one half of its inner surface in a state of the highest inflammation, especially at its pyloric extremity.

Mr. B. thinks the gastric and peritoneal inflammation existed from the time of this man's admission, and "increased so gradually as not to excite the symptoms by which its existence may be generally known." We differ from him *in toto*, and consider it a metastasis of acute rheumatism, which produces the most rapid and destructive ravages on internal organs, if not immediately checked.

CHAP. 5. *On the Effects of Ardent Spirits on the Stomach.*—Mr. B. considers the degree of *irritability* in the iris of an intoxicated man as the best index of the degree of danger. When the iris contracts on the application of light, the danger is not great; but the reverse is a very dangerous sign. On vomiting he depends for a cure. When reaction takes place, the shower bath, venæsection, and purgatives.

"If we can excite full vomiting, we shall save our patient; if we cannot, he will have a better chance of recovery, if we leave him to the operations of nature, than if we bleed him, agitate him, endeavour to make him walk, or in any way disturb him."

We do not entirely agree with Mr. B. in this. We have seen good effects from cold lotions to the head; blisters between the shoulders; acrid purgative glysters, and pediluvia, when no means of rousing the patient, or exciting vomiting, were effectual.

In those brains which our author had an opportunity of inspecting, the vessels were found gorged with blood, and a quantity of serum deposited in the lateral ventricles. No traces of inflammation or other morbid effect could be found in the stomach.

From this till we come to the 13th Chapter, nothing interesting occurs. Here we have Mr. Bedingfield at his old work—Physiology.

"*On the Functions of the Liver.*"—The two cases of *lusus naturæ*, found by Messrs. Abernethy and Lawrence, wherein the *hepatic artery* supplied the place of the *vena portæ* have quite satisfied Mr. B. that we have been all in the dark about the last-mentioned vessel being destined for the secretion of bile! The thousand millions of instances wherein the vena portæ did *not* terminate in the cava, are nothing compared to the *two* instances above related!

Why does not Mr. Bedingfield maintain, on the same principle, that the many *akerious* and *acephalous* instances on record, are sufficient proofs that *hearts* and *heads* are merely accidental, perhaps useless appendages to the human frame, since ganglia and arteries might have served the purpose quite as well.

"To affirm that bile is formed by a vein, is so directly in opposition to the general and known laws of the animal economy, that it is a matter of surprize how such an assertion should have gained almost universal credence." p. 207. Indeed!

But pray, Mr. Bedingfield, does not the vena portæ imitate an artery in no other way than in its secretory office? What does any artery, that the vena portæ does not do? Does it not ramify into smaller and smaller branches *in the direction of the current of blood*, contrary to *all* other veins; and in common with *all* other arteries? Does it not drive its blood through the substance of a gland, what no other vein does, and finally, do not the penicilli of that gland arise from the capillary system of the portæ and hepatic artery? And for what purpose has the vena portæ been deprived of its long established office?

"It appears to me, that the vena portæ is intended to preserve an equilibrium in the circulation of the blood."

That the system of the vena portæ preserves the heart and lungs from being overpowered with blood in the cold stages of fever, and other states of the blood-vessels where violent oscillations in the circulation take place, has been argued by a preceding writer,* without attempting to deprive it of its biliary function. So that even here, our author is as much behind hand, as in his physiology of the brain!

Chap. 14. *Peritonitis.*—The dangerous and insidious nature of this disease is well known. We believe with Mr. B. that evacuations from the vascular system are seldom carried to a proper extent, owing to the symptoms being milder than in many other inflammations, and also to the apparent debility which accompanies them. The violence of pain, and tenderness on pressure are better criteria for estimating the patient's danger, and regulating our own practice, than the state of the pulse. After the vascular action is subdued, a large blister to the abdomen is generally necessary. Chronic peritonitis, the occasional sequela of acute, especially when the depleting system has not been sufficiently acted on, must be treated with leeches, blisters, and small doses of calomel, with an active cathartic twice a week.

Chap. 17. *Ascites.*—Great numbers afflicted with this disease have been admitted into the Bristol Infirmary.

"In a great majority of cases, more advantage was gained from mercurial friction, and an occasional drastic purgative, than from any other remedies."

Our own experience confirms this. A scruple or half a drachm of ung. hyd. was rubbed over the abdomen, till the mouth became affected slightly; while from two to five grains of elaterium were given once or twice a week. In weak habits, much smaller doses of this last medicine was given combined with the squill pill. In robust habits, a quarter of a grain, every intermediate day, was found eminently useful.

---

* *Vide* "Influence of Tropical Climates on European Constitutions." *passim.*

Mr. B. is averse to the operation till it cannot longer be avoided; and he proposes its performance when the system is under the influence of mercury, an influence that may be sufficient to remove a small, though not a large quantity of water. We think this suggestion, by no means an unfeasable one; on the contrary, that it is worthy of trial.

Chap. 19. *Diarrhœa.*—

"For one, two, or three nights in succession, as circumstances may indicate, from three to five grains of calomel with one grain of opium, should be given."

An occasional dose of Castor oil in the morning. Mr. B. saw at least fifty violent cases successfully treated in this manner. We may add, that we have seen fifty times fifty treated on the same plan, or nearly so, with success.

Chap. 20. *Tympanites.*—Active purgatives, and mercurial frictions upon the surface of the abdomen and thighs, more frequently remove this affection than any other medicine.

*Tænia.* Ol. tereb. rectif. was found the best remedy for this worm. An ounce to a delicate female; ℥iß to a robust female or small man; to a robust man ℥ij. Milk was found the best vehicle. The morning fasting the best time for its exhibition.

"The greater distress occasioned by small than by large doses is not confined to the action of ol. tereb. Three grains of calomel will often act as a violent purgative, whereas *a scruple* of it is one of the mildest aperients that can be given." p. 230.

How often we have stated this fact in our Journal, our readers are well aware.

*Lumbrici.* ℞. Pulv. jalap. Pulv. Rhei. āā gr. xv. Submur. hyd. gr. v. fiat pulvis singulis auroris sumendus per tres vel quatuor vices. The same for ascarides.

Chap. 22. *Case.*—Mary Strange, ætat. 29, aborted in December 1810, and lost three pints of blood, after which the catamenia were very irregular. About five months after abortion she complained of pain in the back and loins, which was much aggravated at the catamenial periods. Palpitation of the heart accompanied this pain, with nausea and sickness. Bleeding and blistering produced little relief. February 1811, she had a violent attack, from which time till September she gradually recovered, and was able to go about her ordinary business.

She was soon after, however, seized with violent pain in the left hip, extending across the bowels to the back, and from thence to the pit of the stomach. Great palpitation; faintness; respiration now loud and laboured; again, scarcely perceptible; veins of the head, neck, and upper extremities greatly distended. These paroxysms occurred four or five days in the week, subsiding gradually with hysterical sobbing and crying. November 1811, she had a return of the catamenia, which continued ten or twelve days, profuse and dark coloured. Great tenderness in the hypogastric region. In this state she continued till July 1812, when she was admitted into the hospital, where she died in a few days, a pulsatory motion being felt on pressing the abdomen.

*Sectio Cadaveris.* On opening the abdomen an immense tumor was exposed, occupying almost the entire left side of that cavity. Through the peritonæum which covered it, it looked very much like the uterine surface of a placenta. Upon dividing the peritonæum, the tumor was found to consist of coagulated blood. Six or seven handfuls of this being scooped out, the left kidney presented itself. It occupied nearly the centre of the tumor. The emulgent vessels were elongated; the fore finger passed upon them to their origin, and then directed a little upwards, went immediately into the cavity of the descending aorta. It appeared, that a circular piece of the artery had been removed by the absorbents. Upon the inner surface of the peritonæum no layers of coagulable lymph had been deposited; there was not, in fact, any thing which resembled an aneurismal sac. No disease in the other abdominal viscera.

CHAP. 23. *Nephritis.*—The remedies found most effectual in this disease at the Bristol Infirmary, were—venæsection from a small orifice *ad deliquium:* cupping; leeches; blisters to the lumbar region; warm bath.

Blisters, thinks our author, are not so liable to excite strangury, when applied on the immediate vicinity of the urinary organs, as when they are placed upon parts remote from them. The warm bath produces a powerful determination of blood to the surface of the body, and greatly increases the action of the cutaneous exhalents. Constipation is to be guarded against by gentle aperients. Diuretics are injurious.

We had hoped to conclude our Analysis of this volume in the present Number, but find that we cannot, consistently with our plan of presenting, not merely a sketch, but in some measure, a well defined picture of the medical literature of the day to our readers. It will be seen also, that although we had some *words* with Mr. Bedingfield at the beginning, especially on theoretical and doctrinal subjects, yet our acquaintance has improved, and we shall probably part on very good terms.

